# Regional disparities in Japanese children's mental health profession shortage areas

**DOI:** 10.1002/pcn5.70107

**Published:** 2025-05-07

**Authors:** Masahide Usami, Yoshinori Sasaki, Mayuna Ichikawa, Miki Matsudo, Ayaka Hashimoto, Mutsumi Ohashi, Yui Higashino, Yusuke Kono, Haruna Matsudo, Yuki Nomura, Minjae Ma, Yuuki Sakoh, Maiko Odaka, Kotoe Itagaki, Keita Yamamoto Mhsw, Momoka Takahashi Mhsw, Yuta Yoshimura, Saori Inoue, Masahiro Ishida, Kumi Inazaki, Yuki Hakoshima, Yuki Mizumoto

**Affiliations:** ^1^ Department of Child and Adolescent Psychiatry National Kohnodai Medical Center, Japan Institute for Health Security Ichikawa Japan; ^2^ Department of Psychiatry and Behavioral Science Tokyo Medical and Dental University Tokyo Japan; ^3^ Department of Clinical Psychology Kohnodai Hospital, National Center for Global Health and Medicine Ichikawa Japan; ^4^ Department of Social Work Kohnodai Hospital, National Center for Global Health and Medicine Ichikawa Japan; ^5^ Department of Psychiatry Fukuoka University Fukuoka Japan; ^6^ Department of Child Psychiatry Ehime University Graduate School of Medicine Toon City Japan

**Keywords:** child, mental health, Japan

## Abstract

**Aim:**

This study aims to investigate regional disparities in the distribution of child and adolescent mental health professionals in Japan. It focuses on identifying areas where access to specialists is limited and proposes potential solutions to address these inequalities.

**Methods:**

Data were collected from multiple sources, including government statistics and public reports, on population size, number of specialists, mental health facilities, rates of truancy, bullying, suicides, and child abuse. A cluster analysis using the K‐means method was conducted to categorize all 47 Japanese prefectures into groups based on access to child and adolescent psychiatric care.

**Results:**

Significant regional disparities were found in the distribution of child psychiatry specialists, with a 4.7‐fold difference in student‐to‐specialist ratios across regions. Rural areas exhibited severe shortages of specialists compared to urban areas. Prefectures were classified into three clusters based on their access to mental health resources, with rural areas showing the greatest need for additional support.

**Conclusion:**

There are considerable regional disparities in access to child and adolescent psychiatric care in Japan. To address these, policy measures, such as promoting specialist placement in rural areas, enhancing specialist training programs, and introducing telemedicine, are necessary. These steps can help ensure more equitable access to mental health services nationwide.

## Graphical abstract

Map by prefecture showing the lack of child psychiatric resources.

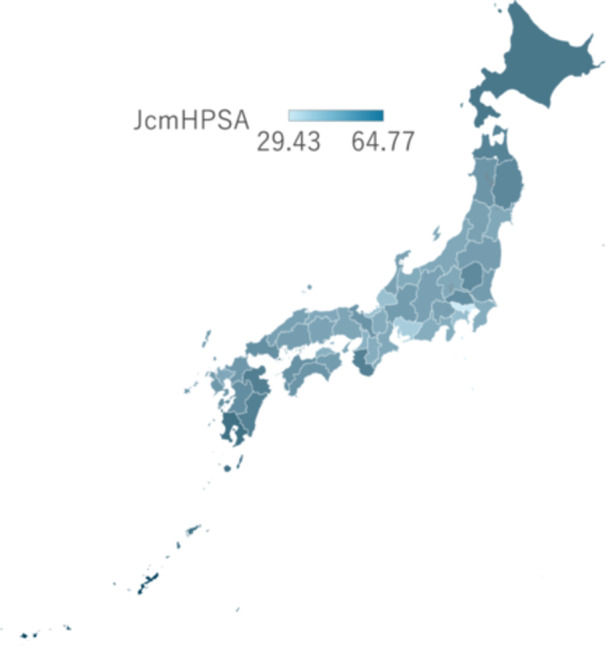

## INTRODUCTION

Child and adolescent psychiatry have a major impact on the mental health of children and adolescents. However, in Japan, the healthcare system in child and adolescent psychiatry is facing various challenges. Since the COVID‐19 pandemic, the severity of these challenges has become more pronounced. For example, in FY2021, the number of elementary and junior high school students who did not attend school reached 240,000, thereby bringing the issues of school refusal and truancy to public attention. This issue may result from environmental changes and restrictions on school life that increased the psychological burden on children.^1^, ^2^

In addition, the number of suicides among those under the age of 18 increased, reaching 514 in FY2022 and 513 in FY2023. This report was higher than previous statistics, indicating the rapidly growing need for psychological support for children and adolescents in Japanese society. In particular, the number of individuals being diagnosed with autism spectrum disorder (ASD) and attention‐deficit/hyperactivity disorder (ADHD) was rapidly increasing.^3^ These children need appropriate medical services for their diagnosis and treatment by child and adolescent psychiatrists.

However, various issues exist in the current system of providing child and adolescent psychiatry care in Japan. According to a survey by the Ministry of Internal Affairs and Communications, the waiting time for an initial consultation for developmental disabilities is a serious problem, with many cases reporting 3 months or longer of waiting. Furthermore, some medical institutions have reported waiting times of up to approximately 10 months, and more than 50 people have been identified on the waiting list. Some institutions have 316 patients on the waiting list for initial consultations, making it difficult for patients to receive appropriate medical care.^4^ This prolonged waiting time can be attributed to the continuing shortage of medical specialists. In Japan, although child and adolescent psychiatrists are concentrated in urban areas, the number of specialists remains low in rural and depopulated areas. For example, in metropolitan areas, such as Tokyo and Osaka, the number of specialists is high, allowing patients to receive medical services relatively early. However, in rural areas, the number of medical specialists is significantly lower, indicating the existence of large regional disparities in access to medical care. Furthermore, behind these medical disparities lies an underdeveloped training system. In Japan, child and adolescent psychiatry is not fully recognized as an independent specialty, which has led to a delay in the introduction of specialized training programs. Training new specialists in rural areas is also difficult; thus, the number of specialists in rural areas remains insufficient.^5^, ^6^

In the United States, health professional shortage areas (HPSAs) are officially designated for areas, specific populations, or facilities where there is a shortage of healthcare professionals. The score plays an important role in the US Healthcare Policy, and it is used to assess equity in access to healthcare by region. HPSAs have three types: geographic HPSAs, which are areas with a shortage of healthcare providers across a specific geographic region; demographic HPSAs, which refer to areas within a specific geographic region with a shortage of providers for specific population groups (e.g., low‐income, homeless, or migrant workers); and institutional HPSAs, which are public or nonprofit healthcare facilities that lack healthcare providers for a specific geographic area or population.

Based on Japan's monoethnic population and universal health insurance system, large regional disparities in access to healthcare are reported according to the 2018 statistics. The child poverty rate reached approximately 13.5%, with one in seven children living in poverty. In addition, poor families are at increased risk of child abuse and mental illness, and children living in areas with difficult access to healthcare have limited opportunities to receive appropriate medical services. Therefore, introducing regional healthcare access indicators to the HPSA is necessary in Japan.

This study aims to clarify regional disparities in Japan and provide evidence to alleviate the shortage of child psychiatry professionals in accordance with the HPSA evaluation methodology in the United States. In particular, the study aims to evaluate the actual state of child and adolescent psychiatry in each region based on various official and publicly available statistical information and results, as well as to make policy recommendations to correct the regional disparities. The results of this study will contribute to the realization of a society in which all children have equal access to necessary medical services.

## METHODS

### Health professional shortage areas

We have reviewed the scientific literature regarding the development and use of the US HPSA score. Studies have demonstrated that the HPSA designation is associated with socioeconomic factors, such as lower income levels and reduced healthcare access in rural areas.^7^, ^8^ Additionally, research has identified limitations in the HPSA methodology, such as the potential exclusion of certain provider types when calculating provider‐to‐population ratios, which may lead to inconsistencies in designation.^9^

Furthermore, studies have shown that HPSA designation has significant public health implications, including lower participation in clinical cancer trials in rural and HPSA‐designated areas.^10^ These findings indicate that while the HPSA methodology provides a useful framework for identifying healthcare shortages, it is not without limitations in terms of comprehensiveness and accuracy.

Based on this literature, we acknowledge that while the HPSA methodology is widely used in policy‐making, its scientific validity is not without criticism.

### Japanese children's mental health professional shortage areas (JcmHPSA) methodology

#### Domains of the JcmHPSA compared to the HPSA

Below is a summary of the major differences. The table compares the methodologies of the US HPSA and the JcmHPSA, highlighting modifications made to adapt the US framework to Japan's unique context.

#### Geographic score

- *HPSA methodology:* Calculates scores based on the population‐to‐provider ratio within a defined geographic area.
- *JcmHPSA methodology:* Evaluates area size and accessibility indices from Japan's Ministry of Land, Infrastructure, Transport, and Tourism.
- *Rationale for modification:* This adjustment addresses Japan's unique geographic and accessibility challenges, ensuring that the scoring reflects actual access to care.

#### Demographic score

- *HPSA methodology:* Considers population characteristics, including poverty levels.
- *JcmHPSA methodology:* Incorporates child‐specific factors, such as poverty rates, truancy rates, and child abuse cases.
- *Rationale for modification:* Focusing on these variables provides a more accurate assessment of child mental health accessibility in Japan, as they directly impact the need for specialized services.

#### Institutional score

- *HPSA methodology:* Assesses the number of healthcare providers and facilities.
- *JcmHPSA methodology:* Utilizes the population‐to‐specialist ratio and counts specialized child psychiatry facilities.
- *Rationale for modification:* Given that child and adolescent psychiatry is not a recognized specialty in Japan, this approach offers a tailored assessment of available resources, accounting for the country's specific healthcare structure.

These modifications ensure that the JcmHPSA methodology accurately reflects Japan's demographic and institutional landscape, providing a more relevant evaluation of child mental health professional shortages.

### Geographic information, demographic data, and social issues

The area (km²) of each prefecture used in this study; the population of elementary and junior high school students; the number of specialists; the number of medical institutions with specialist wards; the number of truancy, bullying, violence, and suicide cases; the number of child mental health specialists; comprehensive access scores; and child poverty rates were collected from information published by government agencies. These data were obtained from the Statistics Bureau of the Ministry of Internal Affairs and Communications' Statistics in Japan 2023; the Ministry of Education, Culture, Sports, Science and Technology's 2022 survey on student guidance issues, such as problematic behavior and truancy among pupils; the website of the Organization of Medical Specialists for Children's Mental Health; and the Ministry of Land, Infrastructure, Transport and Tourism's website on access scores updated January 2024 (Statistics Bureau of the Ministry of Internal Affairs and Communications 2023, Ministry of Education, Culture, Sports, Science and Technology 2022, Organization of Medical Specialists for Children's Mental Health 2024, and Ministry of Land, Infrastructure, Transport and Tourism 2024).

Regarding specialized child and adolescent psychiatric institutions, information on the locations and bed numbers for full member and observer institutions was obtained from the website of the Japanese Council of Child and Adolescent Mental Institutions updated in April 2023 (the Japanese Council of Child and Adolescent Mental Institutions 2024).

In general, the government calculates the “poverty rate” using the “relative poverty rate,” in which national incomes are sorted from highest to lowest, and the income group with less than half of the median income is identified as “poor.” However, for the data used in this study, the poverty rate was calculated by considering “households living on less than welfare income” as “poor.” In 2016, “18.3% of all households and 13.8% of child‐rearing households in Japan were reported living on incomes below the welfare standard,” and this is the latest result by prefecture.^11^

### Rate of suicide

Regarding the number of suicides from national data, 19 were elementary school students, and 123 were junior high school students, with incidence rates of 0.00031% and 0.00198%, respectively. The “number of suicides” refers to the number of people who actually took their own lives and does not include suicide attempts or suicidal thoughts. The estimated number of children who attempted suicide was calculated on the basis of the number of children obtained from the “Results of the 2022 Survey on Student Guidance Issues, Including Problematic Behavior and Truancy of Students” by the Ministry of Education, Culture, Sports, Science and Technology.

### Japanese children's mental health professional shortage areas

The JcmHPSA was developed on the basis of the US HPSA score approach, which assesses the difficulty of accessing healthcare in each region by summing up the scores obtained for the following factors. Higher scores indicate greater difficulty in accessing healthcare in the region (maximum of 45 points). The JcmHPSA was calculated by summing Geographic HPSA, Demographic HPSA, and Institutional HPSA. The JcmHPSA provides an overall assessment of the percentage of child psychiatric professionals in each region, indicating the differences in the shortage of child psychiatric professionals for children in different regions.

#### Geographic HPSA: (maximum of 20 points)

- *Geographic score:* It evaluates the size of an area. The larger the area, the more difficult it is to access physically (maximum 10 points). The prefecture with the lowest population density was given a score of 10.
- *Access score:* It refers to the weekly accessibility index of services by prefecture published by the Ministry of Land, Infrastructure, Transport and Tourism, as well as the overall accessibility index among temporal accessibility index, spatial accessibility index, overall accessibility index, and monetary accessibility index. The most comprehensive accessibility index was adopted. The prefecture with the lowest overall accessibility index was given a score of 10.

#### Demographic HPSA: (maximum 20 points)

- *Child poverty score:* Child poverty is considered a risk for maltreatment and various mental illnesses, and access to healthcare is difficult for economic reasons. A score of 10 was assigned to the prefectures with the highest poverty rates.
- *School refusal score:* The number of school refusals was used as an indicator to reflect social stressors. Regions with a higher number of school refusal cases have a higher social burden and receive a higher score (maximum of 5 points). The region with the highest number of school refusal cases relative to the population was given a score of 5.
- Child abuse score: The number of abuses indicates social stressors. Areas with the highest number of abuses were considered to have a higher social burden and scored higher (up to 5 points). The area with the highest number of abuses relative to the population was scored 5.

#### Institutional HPSA: (maximum 20 points)

- *Population‐to‐specialist ratio score:* It assesses the number of specialists per region, indicating differences in the availability of healthcare facilities in different regions. The number of specialists relative to the region's population was evaluated. The higher the population per specialist, the more difficult it is to access medical care (maximum score of 10). The region with the lowest number of specialists to population was given a score of 5.
- *Specialty facility ratio score:* It assesses the number of specialty facilities relative to the region's population. A score of 10 is given to prefectures with no specialty facilities.

### Statistical methods

In this study, cluster analysis using the K‐means method was conducted to identify regional disparities in child psychiatric care across all prefectures in Japan. This analysis aimed to group similar prefectures based on the characteristics of each region and to provide a basis for policy recommendations. Eight variables were used in cluster analysis: total population of elementary and secondary school students, population density, overall access score, child poverty rate, number of medical specialists, number of specialized hospitals, truancy rate, and number of abuses/children. Each of these variables represents an important aspect of child psychiatric care in the region. These variables were used to provide a comprehensive assessment of the medical resources and socioeconomic context of each region.

First, each variable was standardized to equalize the impact of data with different scales. A Z‐score transformation was used for standardization, where the mean of each variable was set to 0 and the standard deviation was set to 1. Next, K‐means clustering was performed on the standardized data to classify all 47 prefectures into three clusters. The K‐means method is a nonhierarchical clustering technique that classifies data into a predefined number of clusters. In this study, the number of clusters was set to 3 to achieve optimal clustering.

## RESULTS

### Population and distribution of medical specialists by prefecture

Table 1 shows the population, area, number of specialists, number of hospitals with child and adolescent wards, and number of beds for elementary and junior high school students by prefecture.

**Table 1:** Population, specialists, number of specialty hospitals, number of beds by prefecture.

Prefectures	Elementary school students	Junior high school students	Area (km^2^)	Child poverty rate	Total accessibility	Number of specialists	Number of special hospitals	Number of beds	Primary and secondary school students	Students/km^2^	Specialists/km^2^	Students per specialist	Students/hospital	Children/bed
Kagawa	48,312	25,469	1876	12	68	12	1	22	73,781	39	0.0064	6148	73,781	3354
Fukui	38,844	21,261	4190	6	87	9	0	0	60,105	14	0.0021	6678	—	—
Nara	65,223	35,897	3691	12	100	15	0	0	101,120	27	0.0041	6741	—	—
Tottori	28,248	14,473	3507	15	94	5	0	0	42,721	12	0.0014	8544	—	—
Tokushima	33,820	17,563	4147	12	78	6	0	0	51,383	12	0.0014	8564	—	—
Kyoto	121,460	65,857	4612	17	97	20	0	0	187,317	41	0.0043	9366	—	—
Hiroshima	146,474	46,235	8479	15	100	20	1	43	192,709	23	0.0024	9635	192,709	4482
Osaka	420,606	221,249	1905	22	73	65	3	102	641,855	337	0.0341	9875	213,952	6293
Wakayama	43,599	25,652	4726	18	68	7	0	0	69,251	15	0.0015	9893	—	—
Nagano	101,071	54,436	13,562	11	73	15	1	15	155,507	11	0.0011	10,367	155,507	10,367
Toyama	47,142	25,850	4248	6	68	7	0	0	72,992	17	0.0016	10,427	—	—
Okayama	96,766	50,845	7114	16	75	14	1	20	147,611	21	0.002	10,544	147,611	7381
Ishikawa	56,104	30,049	4186	10	73	8	0	0	86,153	21	0.0019	10,769	—	—
Gifu	100,863	54,017	10,621	9	71	14	0	0	154,880	15	0.0013	11,063	—	—
Miyagi	111,733	58,916	7282	15	97	15	2	62	170,649	23	0.0021	11,377	85,325	2752
Yamanashi	38,141	20,564	4465	12	83	5	1	27	58,705	13	0.0011	11,741	58,705	2174
Tokyo	629,794	319,760	2194	10	88	80	3	235	949,554	433	0.0365	11,869	316,518	4041
Kochi	30,993	16,588	7104	19	81	4	1	14	47,581	7	0.0006	11,895	47,581	3399
Niigata	102,435	54,578	12,584	12	97	13	1	40	157,013	12	0.001	12,078	157,013	3925
Shiga	79,680	41,147	4017	9	88	10	0	0	120,827	30	0.0025	12,083	—	—
Ehime	65,092	34,895	5676	17	88	8	0	0	99,987	18	0.0014	12,498	—	—
Aichi	401,886	208,884	5172	11	78	45	4	112	610,770	118	0.0087	13,573	152,693	5453
Mie	88,572	47,484	5777	10	75	10	1	80	136,056	24	0.0017	13,606	136,056	1701
Aomori	53,644	29,042	9645	18	78	6	0	0	82,686	9	0.0006	13,781	—	—
Hyogo	277,810	143,988	8396	15	71	30	3	125	421,798	50	0.0036	14,060	140,599	3374
Gunma	92,532	51,020	6362	10	78	10	1	24	143,552	23	0.0016	14,355	143,552	5981
Yamagata	49,045	26,769	9323	12	71	5	1	15	75,814	8	0.0005	15,163	75,814	5054
Hokkaido	229,096	122,332	83,456	20	75	22	0	0	351,428	4	0.0003	15,974	—	—
Shizuoka	180,451	97,226	7778	11	81	17	2	86	277,677	36	0.0022	16,334	138,839	3229
Fukuoka	279,500	141,464	4986	20	83	25	3	51	420,964	84	0.005	16,839	140,321	8254
Shimane	33,916	17,460	6708	9	71	3	1	26	51,376	8	0.0004	17,125	51,376	1976
Saga	45,035	24,139	2440	11	93	4	1	40	69,174	28	0.0016	17,294	69,174	1729
Miyazaki	58,775	30,890	7735	20	73	5	1	30	89,665	12	0.0006	17,933	89,665	2989
Ibaraki	140,235	75,948	6097	9	73	12	1	36	216,183	35	0.002	18,015	216,183	6005
Kanagawa	448,420	228,368	2415	11	93	35	2	69	676,788	280	0.0145	19,337	338,394	9809
Akita	37,976	21,493	11,612	10	73	3	0	0	59,469	5	0.0003	19,823	—	—
Nagasaki	67,972	35,404	4096	17	78	5	2	35	103,376	25	0.0012	20,675	51,688	2954
Iwate	54,788	29,827	15278	14	68	4	1	18	84,615	6	0.0003	21,154	84,615	4701
Oita	56,677	29,761	6340	14	71	4	0	0	86,438	14	0.0006	21,610	—	—
Okinawa	101,352	50,024	2281	38	68	7	0	0	151,376	66	0.0031	21,625	—	—
Fukushima	85,602	45,531	13783	12	75	6	2	66	131,133	10	0.0004	21,856	65,567	1987
Kagoshima	88,264	45,759	9189	21	75	6	0	0	134,023	15	0.0007	22,337	—	—
Saitama	360,127	186,984	3797	12	68	23	1	30	547,111	144	0.0061	23,787	547,111	18,237
Kumamoto	95,872	49,103	7409	17	97	6	2	61	144,975	20	0.0008	24,163	72,488	2377
Chiba	304,532	158,130	5157	10	99	19	2	73	462,662	90	0.0037	24,351	231,331	6338
Yamaguchi	63,826	33,790	6112	14	75	4	1	30	97,616	16	0.0007	24,404	97,616	3254
Tochigi	94,383	51,274	6408	10	73	5	1	15	145,657	23	0.0008	29,131	145,657	9710

The smallest combined population of elementary and junior high school students is 42,721, whereas the largest combined population is 949,554, with a difference of 22.23 times. The difference in the area of the prefectures has a maximum of 44.49 times. The number of specialists also varies widely, from a minimum of three to a maximum of 80, accounting for a minimum of 6148.4 and a maximum of 29,131.4 elementary and junior high school students per specialist, with a mean and standard deviation of 15,116.16 ± 5616.24. A difference of 4.74 times was observed between the prefectures with the least and highest number of hospitals with child and adolescent wards.

The mean and standard deviation of the number of specialists per km^2^ (persons/km^2^) was 0.004 ± 0.007 persons/km^2^, with a minimum of 0.0003 specialists/km^2^ and a maximum of 0.0365 specialists/km^2^, accounting for a difference of 121.67 times. The number of medical facilities with specialized wards had a minimum of 0 facilities/prefecture and a maximum of four facilities/prefecture, with a mean and standard deviation of 0.89 ± 0.98 facilities/prefecture. Moreover, 17/48 (%) prefectures had no specialized wards with a minimum of 1701 and a maximum of 18,237 children per bed, and the mean and standard deviation was 5109.30 ± 3500.59 children/bed. The difference between the lowest and the highest number of prefectures was 10.72 times greater than that between the lowest and the highest number of prefectures.

### School refusal and bullying in school

Table 2 shows the total number of elementary and junior high school students who refused to go to school and the number of truants per specialist by prefecture. The lowest number of elementary school students who refused to go to school accounted for 441, and the highest accounted for 10,911, with a mean and standard deviation of 2236.43 ± 2338.02. The difference between the two was 24.74. In addition, the lowest number of junior high school students who did not attend elementary school accounted for 877, and the highest accounted for 18,335, with a mean and standard deviation of 4126.30 ± 4070.00. The difference between the two was 20.91 times. The lowest number of elementary school students and the highest number of junior high school students who did not attend school accounted for 1369 and 29,246, respectively, with a mean and standard deviation of 6362.72 ± 6391.10. The difference was 21.91 times greater than that of the lowest number of elementary school students, and 21.36 times greater than that of the lowest number of junior school students. The number of children who do not attend school per specialist was 153 being the lowest and 1033 being the highest, with a mean and standard deviation of 472.15 ± 189.39. The difference between the two was 6.74 times.

**Table 2:** Number of school refusals, suicides, and abused children by prefecture.

Prefectures	Children with school refusal					Presumed suicides				Abused children	
	Elementary school	Junior high school	Elementary and junior high school	Percentage of children with school refusal	Children with school refusal/specialist	Elementary school	Junior high school	Elementary and junior high school	Estimated number of suicides/specialist	Abused children	Abused children/all children
Hokkaido	3729	8591	12,320	3.5%	560.0	0.1	2.4	2.5	11.33%	5930	1.7%
Aomori	611	1638	2249	2.7%	374.8	0.0	0.6	0.6	9.86%	2039	2.5%
Iwate	617	1388	2005	2.4%	501.3	0.0	0.6	0.6	15.19%	1717	2.0%
Miyagi	2066	4122	6188	3.6%	412.5	0.0	1.2	1.2	8.01%	3716	2.2%
Akita	480	1086	1566	2.6%	522.0	0.0	0.4	0.4	14.58%	578	1.0%
Yamagata	685	1388	2073	2.7%	414.6	0.0	0.5	0.5	10.90%	567	0.7%
Fukushima	1049	2497	3546	2.7%	591.0	0.0	0.9	0.9	15.47%	2256	1.7%
Ibaraki	3288	5289	8577	4.0%	714.8	0.0	1.5	1.5	12.89%	4033	1.9%
Tochigi	1563	3604	5167	3.5%	1033.4	0.0	1.0	1.0	20.89%	1627	1.1%
Gunma	1500	2932	4432	3.1%	443.2	0.0	1.0	1.0	10.39%	1897	1.3%
Saitama	4408	9946	14,354	2.6%	624.1	0.1	3.7	3.8	16.58%	18,877	3.5%
Chiba	4618	7703	12,321	2.7%	648.5	0.1	3.1	3.2	16.98%	11,219	2.4%
Tokyo	10,911	18,335	29,246	3.1%	365.6	0.2	6.3	6.5	8.16%	27,798	2.9%
Kanagawa	8076	13,104	21,180	3.1%	605.1	0.1	4.5	4.7	13.32%	23,955	3.5%
Niigata	1621	3138	4759	3.0%	366.1	0.0	1.1	1.1	8.56%	3661	2.3%
Toyama	856	1336	2192	3.0%	313.1	0.0	0.5	0.5	7.52%	1044	1.4%
Ishikawa	1024	1918	2942	3.4%	367.8	0.0	0.6	0.6	7.65%	933	1.1%
Fukui	441	963	1404	2.3%	156.0	0.0	0.4	0.4	4.81%	922	1.5%
Yamanashi	696	1261	1957	3.3%	391.4	0.0	0.4	0.4	8.38%	1451	2.5%
Nagano	2125	3610	5735	3.7%	382.3	0.0	1.1	1.1	7.39%	2697	1.7%
Gifu	1879	3376	5255	3.4%	375.4	0.0	1.1	1.1	7.86%	2684	1.7%
Shizuoka	3340	6303	9643	3.5%	567.2	0.1	1.9	2.0	11.65%	3708	1.3%
Aichi	7408	13,367	20,775	3.4%	461.7	0.1	4.1	4.3	9.47%	9676	1.6%
Mie	1368	2590	3958	2.9%	395.8	0.0	0.9	1.0	9.68%	2408	1.8%
Shiga	1270	2194	3464	2.9%	346.4	0.0	0.8	0.8	8.39%	2187	1.8%
Kyoto	1970	3657	5627	3.0%	281.4	0.0	1.3	1.3	6.71%	5122	2.7%
Osaka	7153	13,651	20,804	3.2%	320.1	0.1	4.4	4.5	6.94%	24,750	3.9%
Hyogo	4961	9642	14,603	3.5%	486.8	0.1	2.9	2.9	9.79%	8971	2.1%
Nara	1145	2229	3374	3.3%	224.9	0.0	0.7	0.7	4.87%	1639	1.6%
Wakayama	671	1260	1931	2.8%	275.9	0.0	0.5	0.5	7.45%	2066	3.0%
Tottori	492	877	1369	3.2%	273.8	0.0	0.3	0.3	5.91%	148	0.3%
Shimane	791	1146	1937	3.8%	645.7	0.0	0.3	0.4	11.87%	332	0.6%
Okayama	1389	2279	3668	2.5%	262.0	0.0	1.0	1.0	7.41%	1220	0.8%
Hiroshima	2759	4678	7437	3.9%	371.9	0.0	0.9	1.0	4.80%	5454	2.8%
Yamaguchi	973	2060	3033	3.1%	758.3	0.0	0.7	0.7	17.22%	688	0.7%
Tokushima	477	1088	1565	3.0%	260.8	0.0	0.3	0.4	5.97%	1039	2.0%
Kagawa	558	1283	1841	2.5%	153.4	0.0	0.5	0.5	4.33%	1152	1.6%
Ehime	891	1837	2728	2.7%	341.0	0.0	0.7	0.7	8.89%	1737	1.7%
Kochi	469	994	1463	3.1%	365.8	0.0	0.3	0.3	8.45%	501	1.1%
Fukuoka	5813	9418	15,231	3.6%	609.2	0.1	2.8	2.9	11.55%	12,332	2.9%
Saga	669	1341	2010	2.9%	502.5	0.0	0.5	0.5	12.30%	1085	1.6%
Nagasaki	981	2100	3081	3.0%	616.2	0.0	0.7	0.7	14.44%	1084	1.0%
Kumamoto	1914	3439	5353	3.7%	892.2	0.0	1.0	1.0	16.70%	2764	1.9%
Oita	816	1887	2703	3.1%	675.8	0.0	0.6	0.6	15.17%	1786	2.1%
Miyazaki	768	1631	2399	2.7%	479.8	0.0	0.6	0.6	12.60%	2019	2.3%
Kagoshima	1256	2565	3821	2.9%	636.8	0.0	0.9	0.9	15.56%	2423	1.8%
Okinawa	2567	3195	5762	3.8%	823.1	0.0	1.0	1.0	14.60%	2585	1.7%

### Number of children who committed suicide and who were abused

Table 2 shows the estimated number of suicides among elementary and junior high school students, their total number, and the estimated number per specialist by prefecture. The lowest estimated number of suicides among elementary school students accounted for 0.009, and the highest accounted for 0.195, with a mean and standard deviation of 0.04 ± 0.04. The difference was 22.30. In addition, the lowest number of junior high school students who committed suicide was 0.29, and the highest was 6.33, with a mean and standard deviation of 1.3554 ± 1.34530. The difference between the two was 22.09. Among elementary and junior high school students, the lowest and highest number of students who committed suicide accounted for 0.2866 and 6.3312, respectively, with a mean and standard deviation of 1.36 ± 1.35. The difference was 22.10‐fold. The number of suicides per specialist was 0.04 being the lowest and 0.21 being the highest, with a mean and standard deviation of 0.11 ± 0.04. The difference was 4.83 times greater than the mean.

Table 2 also shows the number of abused children. The minimum and maximum number of abused children accounted for 148 and 277,984, respectively, with a mean and standard deviation of 4648.45 ± 6583.8. The difference was 187.82 times greater than the mean. The minimum and maximum rate of abused children/all children was 0.35% and 3.86% with a mean and standard deviation of 1.86% ± 0.78%. The difference was 11.13 times greater than the mean.

### Japanese children's mental health professional shortage areas

Table 3 shows the population score, specialty hospital score, truancy score, geography score, abuse score, poverty rate score, and JcmHPSA per specialist.

- *Population‐to‐specialist ratio score:* The minimum and maximum population ratio score per specialist was 2.10 and 10.00, respectively, with a mean value of 5.19 ± 1.90.
- *Specialized facility ratio score:* The minimum and maximum population‐to‐facility scores were 2.00 and 10.00, respectively, with a mean value of 7.96 ± 2.06.
- *Child abuse score:* The minimum and maximum scores were 0.45 and 5.00, respectively, with a mean value of 2.40 ± 1.01.
- *School refusal score:* The minimum and maximum truancy scores were 2.92 and 5.00, respectively, with a mean of 3.90 ± 0.53.
- *Geographical score:* The minimum and maximum geographical scores were 2.00 and 10.00, respectively, with a mean value of 8.87 ± 1.99.
- *Accessibility score:* The minimum and maximum accessibility scores were 2.00 and 10.00, respectively, with a mean of 8.87 ± 1.99.
- *Child poverty score:* The minimum and maximum economic scores were 5.50 and 37.500, respectively, with a mean of 13.7532 ± 5.2503.
- The JcmHPSA provides an overall assessment of the percentage of child psychiatric professionals in each region, indicating the differences in the shortage of child psychiatric professionals for children in different regions. The minimum and maximum values were 22.07 and 42.84, respectively, with a mean of 36.28 ± 3.89.

**Table 3:** The distribution of Japanese children's mental health professional shortage areas (JcmHPSA).

Prefectures			Score							JcmHPSA	Class
	Child poverty rate	Total accessibility	Population ratio score/specialist	Facility score	Non‐school attendance score	Geographic score	Accessibility score	Number of abuses score	Economic scores		
Hokkaido	19.7	74.5	5.5	10.0	4.38	9.90	9.06	2.17	11.52	53	0
Aomori	17.6	77.5	4.7	10.0	3.40	9.80	8.71	3.17	10.29	50	0
Iwate	13.9	67.5	7.3	8.0	2.96	9.87	10.00	2.61	8.13	49	0
Miyagi	15.3	96.5	3.9	6.0	4.53	9.46	6.99	2.80	8.95	43	2
Akita	9.9	72.5	6.8	10.0	3.29	9.88	9.31	1.25	5.79	46	0
Yamagata	12.0	70.5	5.2	8.0	3.42	9.81	9.57	0.96	7.02	44	0
Fukushima	11.6	74.5	7.5	6.0	3.38	9.78	9.06	2.21	6.78	45	0
Ibaraki	8.6	72.5	6.2	8.0	4.96	9.18	9.31	2.40	5.03	45	2
Tochigi	10.4	72.5	10.0	8.0	4.43	9.48	9.31	1.44	6.08	49	0
Gunma	10.3	77.5	4.9	8.0	3.86	9.48	8.71	1.70	6.02	43	0
Saitama	12.2	67.5	8.2	8.0	3.28	6.67	10.00	4.43	7.13	48	1
Chiba	10.4	98.5	8.4	6.0	3.33	7.93	6.85	3.12	6.08	42	2
Tokyo	10.3	87.5	4.1	4.0	3.85	0.00	7.71	3.76	6.02	29	1
Kanagawa	11.2	92.5	6.6	6.0	3.91	3.53	7.30	4.55	6.55	38	1
Niigata	12.0	96.5	4.1	8.0	3.79	9.71	6.99	3.00	7.02	43	2
Toyama	6.0	67.5	3.6	10.0	3.75	9.60	10.00	1.84	3.51	42	0
Ishikawa	10.0	72.5	3.7	10.0	4.27	9.52	9.31	1.39	5.85	44	0
Fukui	5.5	86.5	2.3	10.0	2.92	9.67	7.80	1.97	3.22	38	0
Yamanashi	11.7	82.5	4.0	8.0	4.17	9.70	8.18	3.18	6.84	44	2
Nagano	11.1	72.5	3.6	8.0	4.61	9.74	9.31	2.23	6.49	44	2
Gifu	9.4	70.5	3.8	10.0	4.24	9.66	9.57	2.23	5.50	45	0
Shizuoka	10.8	80.5	5.6	6.0	4.34	9.18	8.39	1.72	6.32	42	2
Aichi	10.9	77.5	4.7	2.0	4.25	7.27	8.71	2.04	6.37	35	1
Mie	9.5	74.5	4.7	8.0	3.64	9.46	9.06	2.27	5.56	43	0
Shiga	8.6	87.5	4.1	10.0	3.58	9.31	7.71	2.33	5.03	42	0
Kyoto	17.2	96.5	3.2	10.0	3.75	9.06	6.99	3.51	10.06	47	2
Osaka	21.8	72.5	3.4	4.0	4.05	2.22	9.31	4.96	12.75	41	1
Hyogo	15.4	70.5	4.8	4.0	4.33	8.84	9.57	2.73	9.01	43	2
Nara	11.7	100.0	2.3	10.0	4.17	9.37	6.75	2.08	6.84	42	2
Wakayama	17.5	67.5	3.4	10.0	3.49	9.66	10.00	3.83	10.23	51	0
Tottori	14.5	93.5	2.9	10.0	4.01	9.72	7.22	0.45	8.48	43	0
Shimane	9.2	70.5	5.9	8.0	4.71	9.82	9.57	0.83	5.38	44	0
Okayama	15.7	74.5	3.6	8.0	3.11	9.52	9.06	1.06	9.18	44	0
Hiroshima	14.9	100.0	3.3	8.0	4.82	9.48	6.75	3.64	8.71	45	2
Yamaguchi	13.5	74.5	8.4	8.0	3.88	9.63	9.06	0.91	7.89	48	0
Tokushima	12.4	77.5	2.9	10.0	3.81	9.71	8.71	2.60	7.25	45	0
Kagawa	11.6	67.5	2.1	8.0	3.12	9.09	10.00	2.01	6.78	41	0
Ehime	16.9	87.5	4.3	10.0	3.41	9.59	7.71	2.23	9.88	47	0
Kochi	18.9	80.5	4.1	8.0	3.84	9.85	8.39	1.35	11.05	47	0
Fukuoka	19.9	82.5	5.8	4.0	4.52	8.05	8.18	3.77	11.64	46	2
Saga	11.3	92.5	5.9	8.0	3.63	9.35	7.30	2.02	6.61	43	0
Nagasaki	16.5	77.5	7.1	6.0	3.73	9.42	8.71	1.35	9.65	46	0
Kumamoto	17.2	96.5	8.3	6.0	4.62	9.55	6.99	2.45	10.06	48	2
Oita	13.8	70.5	7.4	10.0	3.91	9.69	9.57	2.66	8.07	51	0
Miyazaki	19.5	72.5	6.2	8.0	3.34	9.73	9.31	2.89	11.40	51	0
Kagoshima	20.6	74.5	7.7	10.0	3.56	9.66	9.06	2.32	12.05	54	0
Okinawa	37.5	67.5	7.4	10.0	4.76	8.47	10.00	2.19	21.93	65	0

### Cluster analysis

In this study, cluster analysis was conducted for all 47 prefectures in Japan using the K‐means method. To determine the optimal number of clusters, we evaluated several statistical indices, including the Within‐Cluster Sum of Squares (WSS), Silhouette Score, Calinski–Harabasz Index (CH Index), and Davies–Bouldin Index (DBI). The Elbow Method suggested that a three‐cluster solution might be appropriate based on the inflection point of the WSS curve. However, additional validation metrics indicated otherwise:

- Silhouette Score was maximized at *k* = 2 (0.52), suggesting that a two‐cluster solution provided the best‐defined grouping.
- CH Index, which measures between‐cluster variance relative to within‐cluster variance, peaked at *k* = 2, further supporting a two‐cluster model.
- DBI, which evaluates cluster compactness and separation (lower values indicate better clustering), reached its minimum at *k* = 2, confirming the statistical robustness of this classification.

Based on these results, we selected two clusters as the optimal classification for characterizing disparities in child and adolescent psychiatric care resources across Japan.

For validation, an analysis of variance (ANOVA) was conducted to assess the differences among the clusters, yielding a statistically significant difference for each variable (*F* = 15.34, *p* < 0.001).

### Cluster characteristics

- Cluster 1 (Urban Regions with High Accessibility and Resources)

○ Includes Saitama, Tokyo, Kanagawa, Aichi, and Osaka Prefectures.
○ High concentration of mental health specialists with a specialist‐to‐population ratio of approximately 10.00 per 100,000 children (JcmHPSA score: 29.43–42.84).
○ Greater availability of specialized mental health facilities and higher accessibility scores.

- Cluster 0 (Rural Regions with Insufficient Resources)

○ Includes Hokkaido, Aomori, Iwate, Akita, Yamagata, Fukushima, Tochigi, Gunma, Toyama, Ishikawa, Fukui, Gifu, Mie, Shiga, Wakayama, Tottori, Shimane, Okayama, Yamaguchi, Tokushima, Kagawa, Ehime, Kochi, Nagasaki, Oita, Miyazaki, Kagoshima, and Okinawa prefectures.
○ Severe shortage of specialists, with a specialist‐to‐population ratio of only 2.11 per 100,000 children (JcmHPSA score: 42.85–64.77).
○ Lower accessibility scores and fewer specialized child psychiatric facilities, making access to care more challenging.

**Figure 1:**
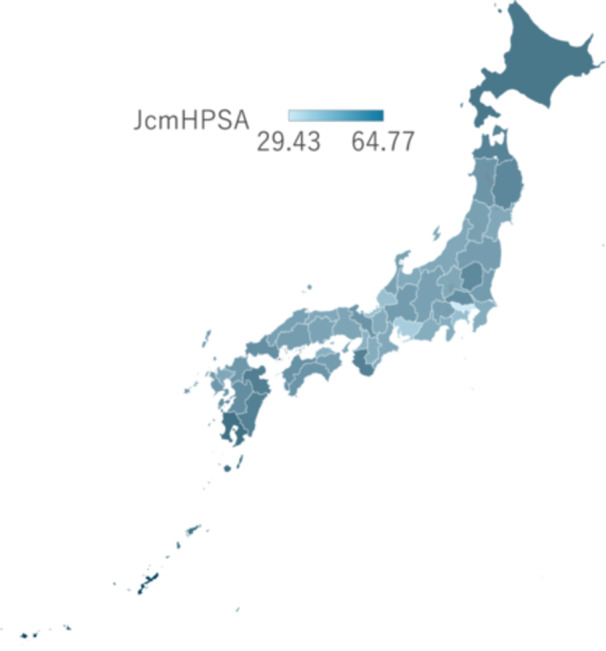
The distribution of Japanese children's mental health professional shortage areas (JcmHPSA) scores across prefectures. Darker colors indicate higher JcmHPSA scores, signifying a greater shortage of child and adolescent psychiatric professionals.

Figure 1 illustrates the distribution of JcmHPSA scores across prefectures. Darker colors indicate higher JcmHPSA scores, signifying a greater shortage of child and adolescent psychiatric professionals. The figure clearly highlights the disparity between well‐resourced urban areas and rural regions facing critical shortages in mental health care.

## DISCUSSION

In clarifying regional disparities in child psychiatric care in Japan, data on the density of specialists by prefecture, prevalence rates of psychiatric disorders among elementary and junior high school students, truancy rates, suicide rates, and the number of child abuse cases were collected and analyzed in this study. The results revealed a serious shortage of medical specialists, especially in rural areas, and significant differences in having access to medical care by region.

### Status of regional disparities

First, significant differences in the number of child psychiatrists were observed among the prefectures. Regarding the number of primary and secondary school students per specialist, a difference of approximately 4.7 times was found between the lowest and highest regions. In the number of specialists per area, a difference of more than 121 times was found between the highest and lowest regions, which indicates difficulty in accessing medical care in rural areas. These differences reflect the shortage of medical resources in rural areas and its impact on the quality and delivery of medical services.

### Regional classification using cluster analysis

In this study, cluster analysis was used to classify the 47 prefectures into three groups and to clarify the distribution of medical resources and socioeconomic background based on the characteristics of each region. This cluster analysis statistically clarified the distribution of medical resources and the socioeconomic background of each region; thus, making specific policy recommendations on issues unique to each region is feasible.

#### Urban areas with sufficient resources

This group includes Tokyo, Osaka, Aichi, Kanagawa, and Saitama Prefectures, which have a high concentration of medical resources and a higher number of child psychiatrists per capita.

The specialist‐to‐population ratio is approximately 10.00 per 100,000 children, significantly higher than in rural and under‐resourced areas. Accessibility scores are high, and specialized mental health facilities are more readily available.

However, demand for mental health services remains high, necessitating further specialist recruitment and service expansion.

#### Rural and under‐resourced areas

This group includes Hokkaido, Aomori, Iwate, Akita, Yamagata, Fukushima, Tochigi, Gunma, Toyama, Ishikawa, Fukui, Gifu, Mie, Shiga, Wakayama, Tottori, Shimane, Okayama, Yamaguchi, Tokushima, Kagawa, Ehime, Kochi, Nagasaki, Oita, Miyazaki, Kagoshima, and Okinawa Prefectures. These regions face significant shortages in child psychiatric care resources.

The specialist‐to‐population ratio is only 2.11 per 100,000 children, far lower than in urban areas with sufficient resources. JcmHPSA scores in this cluster range from 42.85 to 64.77, indicating severe shortages. Many of these prefectures lack specialized child psychiatry facilities, making access to care difficult.

Policies should focus on increasing specialist recruitment, expanding telemedicine, and redistributing medical resources to reduce regional disparities.

### Policy recommendations to eliminate regional disparities

#### Promoting the regional placement of specialists

In rural areas, cluster 0 regions, government policies must be established to provide financial incentives and support for working in rural areas and to promote the placement of specialists. Such policies will promote the retention of specialists and improve access to healthcare in rural areas.

#### Enhanced education and training of specialists

In Cluster 1 and Cluster 2 regions, specialist education and training programs must be enhanced to meet future demand. Training specialists who can provide healthcare in accordance with regional characteristics is important, which includes expanding training programs and introducing curricula tailored to the healthcare needs of each region.

#### Use of telemedicine

The introduction of telemedicine is essential in Cluster 0 areas to improve geographical inaccessibility. Telemedicine can be used to provide quality healthcare services in areas where there is a shortage of specialist doctors. This recommendation will reduce regional disparities in access to healthcare and improve the quality of healthcare services in rural areas.

#### Policies tailored to the needs of each cluster

Establishing region‐specific policies in accordance with the characteristics of each cluster is also important. Cluster 1 requires policies to improve the quality of healthcare, whereas Cluster 2 requires the optimization and efficient use of existing healthcare resources. Cluster 0 requires immediate measures to address the shortage of medical specialists, for example, the introduction of telemedicine and a system of dispatching medical specialists.

#### Regular monitoring of healthcare access indicators

Based on the JcmHPSA and other indicators used in this study, the healthcare access situation in each region should be regularly monitored. In addition, policies should be reviewed, and new measures should be introduced as necessary. This recommendation will enable a rapid response to the healthcare needs of each region and improve the quality of child and adolescent psychiatry nationwide.

### Limitations

This study has several limitations. First, the different years of data used in this study may not fully reflect the most recent healthcare situation. In particular, the lack of an assessment of the outpatient care system caused difficulty in obtaining a detailed picture of the quality and access to outpatient healthcare services in different regions. Additionally, while the JcmHPSA methodology was adapted from the US HPSA framework, it has not been scientifically validated for assessing child and adolescent mental health service shortages in Japan. The selection of variables and scoring criteria was based on available data and expert consensus, rather than a rigorously tested model. As a result, the computed JcmHPSA values may have inherent limitations in accurately reflecting healthcare access disparities. Future studies should aim to validate the methodology and refine the weighting of variables based on empirical evidence. Furthermore, as the study focused on regional disparities within Japan, comparisons with other countries were not made. Consequently, the extent to which issues related to child and adolescent psychiatric care in Japan are prominent internationally cannot be assessed. Given these limitations, future research must ensure consistency of data, comparative studies with other countries, and methodological improvements to enhance the robustness of the JcmHPSA scoring system.

## CONCLUSION

This study reveals that significant regional disparities exist in access to child mental health specialists in various prefectures across Japan. There is a shortage of specialists in rural prefectures, limiting access to appropriate medical care. Thus, promoting the regional allocation of specialists, strengthening education and training of specialists, and utilizing telemedicine are important. Through these policies, homogeneous healthcare services can be provided nationwide.

## AUTHOR CONTRIBUTIONS

Masahide Usami designed this study and wrote this manuscript. Yoshinori Sasaki, Mayuna Ichikawa, Miki Matsudo, Mutsumi Ohashi, Yui Higashino, Yusuke Kono, Haruna Matsudo, Yuki Nomura, Minjae Ma, Yuuki Sakoh, Maiko Odaka, Kotoe Itagaki, Keita Yamamoto, Momoka Takahashi, Yuta Yoshimura, Saori Inoue, Masahiro Ishida, Kumi Inazaki, Yuki Hakoshima, and Yuki Mizumoto collected and discussed this review.

## CONFLICT OF INTEREST STATEMENT

The authors declare no conflicts of interest.

## ETHICS APPROVAL STATEMENT

This study is an analysis of publicly available administrative data and does not require ethical review.

## PATIENT CONSENT STATEMENT

N/A.

## CLINICAL TRIAL REGISTRATION

N/A.

## Data Availability

This study used only public data that anyone can access via the Internet.
